# Towards an Efficient Generalization of the Online Dosage of Hydrogen Peroxide in Photo-Fenton Process to Treat Industrial Wastewater

**DOI:** 10.3390/ijerph182413313

**Published:** 2021-12-17

**Authors:** Xiangwei Yu, Alejandro Cabrera-Reina, Moisès Graells, Sara Miralles-Cuevas, Montserrat Pérez-Moya

**Affiliations:** 1 Chemical Engineering Department, Escola d’Enginyeria de Barcelona Est (EEBE), Universitat Politècnica de Catalunya, Av. Eduard Maristany, 16, 08019 Barcelona, Catalonia, Spain; xiangwei.yu@upc.edu (X.Y.); moises.graells@upc.edu (M.G.); 2 Programa Institucional de Fomento a la Investigación, Desarrollo e Innovación (PIDi), Universidad Tecnológica Metropolitana, Santiago 8940000, Chile; 3 Plataforma Solar de Almería-CIEMAT, Ctra Senés km 4, 04200 Taverns, Almeria, Spain; sara.miralles@ual.es

**Keywords:** photo-Fenton process, hydrogen peroxide dosage, dissolved oxygen, control, wastewater, paracetamol, sulfamethazine

## Abstract

This work addresses the dosage of H_2_O_2_ in photo-Fenton processes and the monitoring of Dissolved oxygen (DO) that can be used to drive the dosage of H_2_O_2_. The objective of this work is to show that a smarter monitoring of a process variable such as DO (for which on-line measurement can be inexpensively obtained) enables the proposal and implementation of efficient dosage strategies. The work explores the application of a recent proposed strategy consisting of: (i) initial H_2_O_2_ addition, (ii) continuous H_2_O_2_ addition until a DO set up is reached, and (iii) automatic H_2_O_2_ addition by an on-off control system based on DO slope monitoring, and applies it to the treatment of different individual contaminants and their mixtures (paracetamol and sulfamethazine). The assays performed following this dosage strategy showed improved values of TOC removed per H_2_O_2_ consumed. For the case of sulfamethazine, this improvement increased up to 25–35% with respect to the efficiency obtained without dosage. Furthermore, a deeper analysis of the results allowed detecting and assessing the opportunity to redesign the dosage scheme and reduce its complexity and the number of control parameters. The promising results obtained are discussed in regard of future research into further increasing the simplicity and robustness of this generalized control strategy that improves the applicability of the photo-Fenton process by reducing its operating costs and increasing automation.

## 1. Introduction

Since the early work by Henry Fenton in the 1890s, the study of the application of Fenton and photo-Fenton processes to the degradation of refractory organic pollutants has developed increasing attention, especially over the last decades. Particularly, the Fenton process is an effective method that consists of the reaction between Fenton reagents, Fe^2+^ (catalyst) and hydrogen peroxide (H_2_O_2_), yielding hydroxyl radical (HO⦁) and Fe^3+^ (Reaction 1). Due to the low reaction rate between Fe^3+^ and H_2_O_2_, the Fenton reaction is boosted with UV radiation (Reaction 2), improving the continuous production of HO⦁, [1]. This highly reactive species unselectively reacts with organic matter (M), producing organic intermediates and, eventually, its complete mineralization (Reaction 3) when adequate operation conditions are selected.

(1) $$Fe^{2+}+H_{2}O_{2}\to Fe^{3+}+HO^{-}+HO⦁$$

(2) $$Fe^{3+}+H_{2}O+\mathrm{hv}\;\to Fe^{2+}+HO⦁+H^{+}$$

(3) $$M+HO⦁\to M_{1}+HO⦁\;\to M_{2}+HO⦁\;\to \ldots \ldots \ldots \to CO_{2\;}+H_{2}O\;$$

At the same time, HO⦁ suffer parallel reactions:

(4) $$HO⦁+HO⦁\to \;H_{2}O_{2}$$

(5) $$H_{2}O_{2}+HO⦁\to HO_{2}{}^{•}+H_{2}O_{\;}$$

(6) $$2HO_{2}{}^{•}\to \;H_{2}O_{2}+O_{2}{}_{\;}$$

Reactions from 4 to 6 are considered unproductive because these consume the HO⦁ at a rate higher than that of organic matter oxidation. Due to the second-order nature of (Reaction 4), if HO⦁ is produced at too high concentration counterproductive reactions are favored which causes the process performance to drop [2]. This poses a trade-off between not limiting the decontamination process due to a lack of H_2_O_2_ and not reaching H_2_O_2_ excess, consequently, adequately dosing H_2_O_2_ along the reaction span arises as an extremely difficult optimization problem that has been barely studied, which is out of the scope of this work. [3,4].

For batch operation, which is the research mainstream, a single addition of H_2_O_2_ at the beginning of the reaction is known to be inefficient [5,6]. During decades, the impact of the Fe^2+^/H_2_O_2_ ratio on process performance was recognized as a critical parameter [7] and was widely studied in the literature [1], nevertheless, the search for a convenient constant ratio is limiting [8]. Such a value should actually be revised according to the variable concentrations caused by process dynamics [4,9]. In this context, the sequential addition of H_2_O_2_ along the reaction span has been reported to enhance mineralization [10,11], furthermore, continuous automatic dosage has also been studied, with positive results [12]. However, the determination of an adequate dosage cannot be limited to a pre-fixed recipe (open-loop control), but it should ideally consider some process feed-back (closed-loop control) to adapt to process disturbances and or model mismatch in a robust way [13]. It is important to stress that the H_2_O_2_ dosage strategy is extremely significant for photo-Fenton process competitiveness because the H_2_O_2_ is the most expensive reagent; consequently, reducing its consumption is key to reducing its operation cost [14].

A first-principles dynamic model mathematically describing the kinetics and evolution of the chemical species involved in this process would allow determining in advance the H_2_O_2_ dosage profile satisfying any given criterion. Lacking such a reliable model, a convenient H_2_O_2_ addition scheme is the on-line adaptation of the dosage based on inferred information about the current status of the process dynamics. This feedback mechanism requires selecting a practical, affordable and informative on-line measurement. Different variables, such as Redox potential [15], have been explored; nevertheless, dissolved oxygen (DO) has demonstrated to be efficient and useful, as well as conceptually consistent with the kinetic model (Reaction 6) [16]. Recently, it was reported that the use of the derivative of the online DO measurement allows providing a significant improvement in process performance [13]. The proposed H_2_O_2_ dosage methodology consists of a hybrid strategy including an initial single H_2_O_2_ addition (open-loop) followed by a continuous addition until an appropriate DO level is attained. Finally, H_2_O_2_ inflow is carried out on a simple on-off control system to keep the DO slope [13] between selected upper and lower bounds (closed-loop). Although proving the concept [13], the evidence and the assessment were limited to samples of synthetic water of a single compound at a fixed concentration (Paracetamol, PCT, 100 mg L^−1^), a limitation also shared by other works [8,16]. Therefore, this work addresses the generalization and improvement of such H_2_O_2_ dosage methodology by extending its validation to different individual pollutants as well as pollutant mixtures, different initial concentrations and different water matrixes.

While the study of the monitoring of DO as a means to provide feedback to the H_2_O_2_ dosage could be performed using a variety of organic compounds, Paracetamol (PCT) and Sulfamethazine (SMT) were selected because of the number of publications addressing their oxidation by means of the photo-Fenton process. Specifically, paracetamol (PCT) is used as a model pollutant in many previous related studies [8,13,16] due to its large consumption worldwide. Seemingly, Sulfamethazine (SMT) is another pharmaceutical product derived from sulfamethoxazole included in the EU Watch List. The concentrations for PCT and SMT were selected assuming wastewaters characterized by high concentration levels of contaminants, total organic carbon (TOC), and chemical oxygen demand (COD), such as pharmaceutical or hospital wastewaters. Thus, the concentration ranges adopted for these contaminants are inspired by the works by Dalgic et al. (2017) [17], who showed that the Fenton process can be an effective pre-treatment of a real paracetamol wastewater of the pharmaceutical industry characterized by a PCT concentration between 37 and 294 mg L^−1^ and Roshanfekr Rad et al. (2015) [18], who investigated the use of the photo-Fenton process in industrial applications and addressed phenol and paracetamol concentrations ranging between 20 and 100 mg L^−1^.

Finally, the possibility of simplifying the dosage strategy by decreasing the number of recipe parameters (inputs) is finally discussed in light of the results.

## 2. Materials and Methods

### 2.1. Reagents

Paracetamol (98% purity), from now on PCT, and Sulfamethazine (99% purity), from now on SMT, were purchased from Sigma-Aldrich, while hydrogen peroxide (33% *w/v*) was purchased from Panreac. The catalyst, added as heptahydrate ferrous sulphate (FeSO_4_∙7 H_2_O), was provided by Merck. Sulfuric acid (95%) used to adjust the pH was provided by Fisher. H_2_O_2_ was determined following the ammonium metavanadate (NH_4_VO_3_ 98.5%) which was obtained from Fisher. Distilled water (DW) was used as water matrix in most of the experiments while the assays carried out to assess water matrix effect were done in natural water (NW).

### 2.2. Pilot Plant

The pilot plant, with a total reaction volume of 15 L, consisted of a glass tube, photoreactor (1.5 L, Table 1) and a reservoir tank (13.5 L) in which a centrifugal pump (Iwaki Magnet Pump, MD-30RZ-220, 1-16HP-220V) operated at a flow rate of 12 L min^−1^ was used to recirculate water assuring the complete mixing of the system.

The incident photon power, E = 3.36 × 10^−4^ Einstein min^−1^ (300 and 420 nm) is provided by a Philips Actinic BL TL 36 W/10 1SL lamp (UVA-UVB). This value was measured using potassium ferrioxalate actinometry. The pilot plant includes on-line measurement sensors for pH (Hamilton Polilyte HTVP 120), temperature and DO/DO slope (Hamilton Oxysens) monitoring. The automatic dosage of H_2_O_2_ is done through a peristaltic pump (Watson Marlow, OEM 313 24V) controlled by a PLC program (Siemens SIMATIC S7-1200) that is managed by InTouchR^®^ software (plant SCADA system). A complete description of the pilot plant can be found elsewhere [13].

### 2.3. Analytical Methods

The decontamination process was monitored through total organic carbon (TOC) concentration, which was measured by a TOC (TOC-VCSH/CSN Shimadzu; Kyoto, Japan) analyzer. Duplicate TOC measurements were always performed. The ammonium metavanadate spectrophotometric method developed by [19] was used to determine H_2_O_2_ concentration (Lambda 365 UV/Vis spectrophotometer, Perkin Elmer, United States).

### 2.4. Experimental Procedure

All the photo-Fenton experiments were done as follows: 15 L of the corresponding type of water were loaded in the system, afterwards, the pollutant was added. With respect to the pollutant, the following options have been assessed: (i) 100 mg L^−1^ of PCT (63 mg TOC L^−1^), (ii) 200 mg L^−1^ of PCT (126 mg L^−1^ of TOC), (iii) 50 mg L^−1^ of PCT and 61.5 mg L^−1^ of SMT (63 mg L^−1^ of TOC, where each pollutant provided half of the total TOC concentration) and (iv) 123 mg L^−1^ of SMT (63 mg L^−1^ of TOC). Then, H_2_SO_4_ was used to set the pH at 2.8 and the selected concentration of pre-dissolved catalyst was added. Recirculation was maintained for 10 min and the first sample was taken for initial iron and TOC measurements. Finally, the UV lamp was switched and H_2_O_2_ addition started simultaneously to trigger the decontamination process.

**Figure 1:**
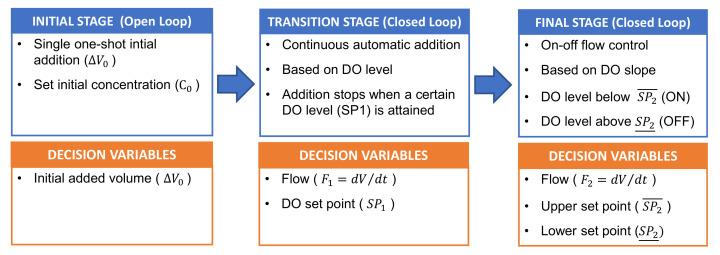
Scheme illustration of the H_2_O_2_ dosage scheme: Initial addition, Constant flow and On-Off control.

Experiments were performed based on the proposed hybrid H_2_O_2_ dosing strategies by Yu et al., 2021 [13], Figure 1:

I. Initial stage (open-loop): Single initial addition at the beginning of the assay. The initial H_2_O_2_ concentrations are selected as a percentage of the H_2_O_2_ stoichiometric concentration (i.e., the stoichiometric concentration for 100 mg L^−1^ of PCT is 472 mg L^−1^; for 123 mg L^−1^ of SMT it is 631 mg L^−1^),
II. Transition stage (close-loop): Continuous automatic addition of H_2_O_2_ from the beginning of the assays at a pre-fixed flow rate of 0.287 mL min^−1^. This addition is maintained until attaining the set DO level. The previously proposed DO set point is 4 mg L^−1^.
III. Final stage (closed-loop): after the transition stage, automatic H_2_O_2_ addition (0.287 mL min^−1^ prefixed flow rate) is based on an on-off controller depending on the DO slope value. In general, the H_2_O_2_ addition is turned off when the DO slope reaches a maximum threshold of 0.2 mg L^−1^ min^−1^, while it is turned on when the DO slope falls below a minimum threshold of 0.1 mg L^−1^ min^−1^.

### 2.5. Experiments Codification

The codification in each experiment aims to allow an easy and fast understanding of the selected dosage strategy. It was used as follows:

I. Pollutant: codification starts with the information about the pollutant or pollutants mixture present in the wastewater by including the abbreviation of the compound preceded by a number that refers to its initial concentration. By way of illustration, PCT at 100 mg L^−1^ corresponds to 1PCT (63 mg L^−1^ of TOC), SMT at 123 mg L^−1^ corresponds to 1SMT (63 mg L^−1^ of TOC) and a mixture of SMT and PCT at 50% each corresponds to 0.5SMT+0.5PCT (also 63 mg L^−1^ of TOC).
II. Initial stage: since initial additions were based on theoretical H_2_O_2_ stoichiometric amounts (coded as S), this information was coded accordingly. By way of illustration, an addition corresponding to 40% of the stoichiometric amount of the pollutant concentration was named 0.4S, which was added after the codification related to the nature and concentration of the pollutant.
III. Transition stage: the DO set point that marks the stop of the H_2_O_2_ continuous addition is then added to the codification. By way of illustration, an initial addition corresponding to 40% stoichiometric amount of PCT at 100 mg L^−1^ using 4 mg L^−1^ of DO as set point to stop the continuous addition was codified as 1PCT_0.4S_DO4. For special assays in which continuous addition during transition stage was never stopped the codification was CA instead of DO4. If the transition stage was not carried out, no codification was added.
IV. Final stage: the selected codification consisted in the addition of the minimum and maximum bounds of the DO slope used for the automatic start and stop of the H_2_O_2_ dosage. By way of illustration, an initial addition corresponding to 40% stoichiometric amount of PCT at 100 mg L^−1^ using 4 mg L^−1^ of DO as set point to stop the continuous addition and 0.2 mg L^−1^ min^−1^ and 0.1 mg L^−1^ min^−1^ as high and low DO slope thresholds of the on-off control system, respectively, was codified as 1PCT_0.4S_DO4_L0.1_H0.2.
V. Iron concentration: 20 mg L^−1^ of iron were used for most of the experiments; however, the catalyst concentration was doubled in a few assays. However, in this last case codification about iron concentration was added at the end (2Fe).

## 3. Results

Different sets of experiments were planned to assess the performance of the dosage strategy under different adjustments and for different substances and mixtures.

The assays are summarized in Table 2. All measurements were duplicated. The two time-series produced for each of the assays showed very high correlation (R^2^ > 0.99) and the TOC residuals obtained were all distributed with a mean of 0.06 mg·L^−1^, a standard deviation of 1.5 mg·L^−1^, a maximum value of 3.35 mg·L^−1^ and a maximum variability (residual value/mean value) of 4.26%. These values are within the reported accuracy (0.5 to 10 mg L^−1^) and variability (approx. 5%) of the analytical method [20]. Once the repeatability and consistency of the measurements are confirmed, average values will be presented from here on.

Assays will be discussed in terms of various outcomes, such as the evolution of the concentration of the different species, and will be quantitatively assessed in terms of the efficiency in which the reactants are used to achieve the mineralization of the organic load (mg TOC removed per mg H_2_O_2_ used). Another performance indicator considered along with this work is the H_2_O_2_ concentration in solution during the experiments, which should be ideally kept within the 50–100 mg L^−1^ range to minimize side reactions.

### 3.1. Water Matrix Effect on Dosage Strategy: Distilled Water vs. Natural Water

Water matrix constituents present a significant impact, in general, on advanced oxidation processes [21] and, particularly, on the photo-Fenton process [22]. The dosage strategy previously proposed [13] and adopted in this work was preliminarily evaluated under conditions closer to the treatment of actual industrial wastewaters by changing the water matrix from DW to NW (Figure 1). Consequently, conductivity, which is a good indicator of the water matrix inorganic content, increased from 1.5 µS cm^−2^ to 1010 µS cm^−2^. This is significant, since the presence of inorganic ions in solution may have a negative effect on the mineralization rate, mainly due to the complexation of the inorganic ions with iron species in solution and the scavenging of HO•, generating other less reactive radicals [23].

**Figure 2:**
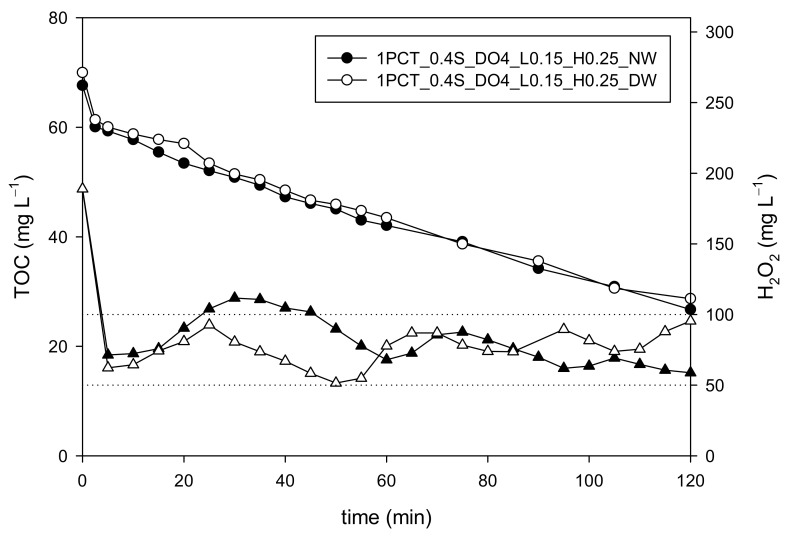
Comparison of TOC (round symbols) and H_2_O_2_ (triangular symbols) concentration profiles when the 0.4S_DO4_L0.15_H0.25 dosage strategy is applied for the treatment of 100 mg L^−1^ PCT (63 mg L^−1^ TOC) contaminated wastewater in NW and DW by photo-Fenton process.

The results (Figure 2) obtained for the initial stage (single one-shot addition of H_2_O_2_) showed negligible differences between NW and DW experiments. For the transition stage (continuous H_2_O_2_ addition until DO achieving the set-up value, 4 mg L^−1^ based on previous studies [13]), the oxygen production was revealed to increase with a higher inorganic load in the water matrix, while the DO set point was reached for NW (20.5 min) 5 min earlier than for DW (25.5 min). Immediately after the transition stage, the continuous addition of H_2_O_2_ is stopped and the automatic addition (on-off control system based on the DO slope signal) is started. The use of 0.15 mg O_2_ L^−1^ min^−1^ and 0.25 mg O_2_ L^−1^ min^−1^ as low and high set points to start and stop H_2_O_2_ addition, respectively, is justified based on previous results [13]. During this last stage, the resulting average H_2_O_2_ concentrations in the reaction bulk were 79.8 ± 17 mg L^−1^ (DW) and 76.8 ± 12 mg L^−1^ (NW).

### 3.2. Validating the Dosage Strategy with Different Pollutant Concentrations

Industrial wastewater characteristics are highly dependent on the type of industry; indeed, within the same type of activity, the organic load depends on the source and time. Hence, to validate the dosage strategy in front of these changes, the PCT initial concentration was increased from 100 mg L^−1^ to 200 mg L^−1^, which corresponds to a TOC increase from 63 mg L^−1^ to 126 mg L^−1^.

**Figure 3:**
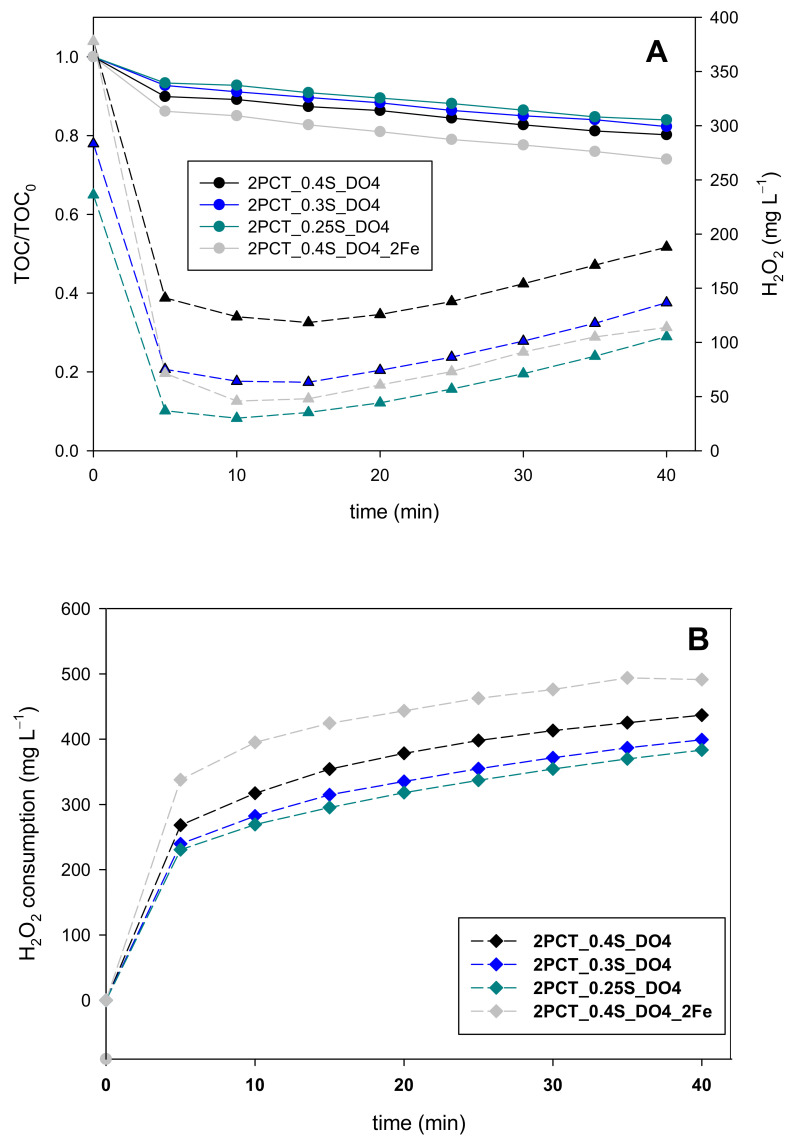
TOC and H_2_O_2_ concentration profiles - round and triangular symbols, respectively - (**A**) and H_2_O_2_ consumption curves (**B**) obtained using 2PCT_0.25S_DO4, 2PCT_0.3S_DO4, and 2PCT_0.4S_DO4 dosage strategies, including an additional assay doubling the iron concentration (2PCT_0.4S_DO4_2Fe).

Double PCT initial concentration decreased the H_2_O_2_ concentration from 378 mg L^−1^ (0.4S, 40% of the stoichiometric concentration) to a minimum value of 118 mg L^−1^ after 15 min (Figure 3A. Then, H_2_O_2_ slowly accumulated in the reactor up to 180 mg L^−1^ after 39 min of reaction time, when DO reached 4 mg L^−1^. These values are above the desired concentration range, which has been ideally set between 50 mg L^−1^ and 100 mg L^−1^.

In this way, the H_2_O_2_ concentration was beyond the 100 mg L^−1^, the value accepted in this work and the literature [14,24], during the whole assay. This suggests that the continuous addition of H_2_O_2_ could be stopped earlier, ideally from the beginning. It is significant that this option would involve reducing the time of the transition stage to zero, which may indicate that the transition stage could be bypassed or removed.

### 3.3. Modifying the Wastewater Organic Matter Nature

#### 3.3.1. Paracetamol and Sulfamethoxazole Mixtures

The 1PCT_0.4S_DO4 strategy tested in 100 mg L^−1^ of PCT (63 mg of TOC L^−1^) was also tested in a mixture containing both PCT and SMT. The new solution was prepared so that each pollutant contributed one half (31.5 mg L^−1^) of the TOC concentration (i.e., 50 mg L^−1^ of PCT and 61.5 mg L^−1^ of SMT). The corresponding assay is coded 0.5PCT_0.5SMT_0.4S_DO4 and it was compared with 1PCT_0.4S_DO4 and SMT_0.4_DO4 assays.

**Figure 4:**
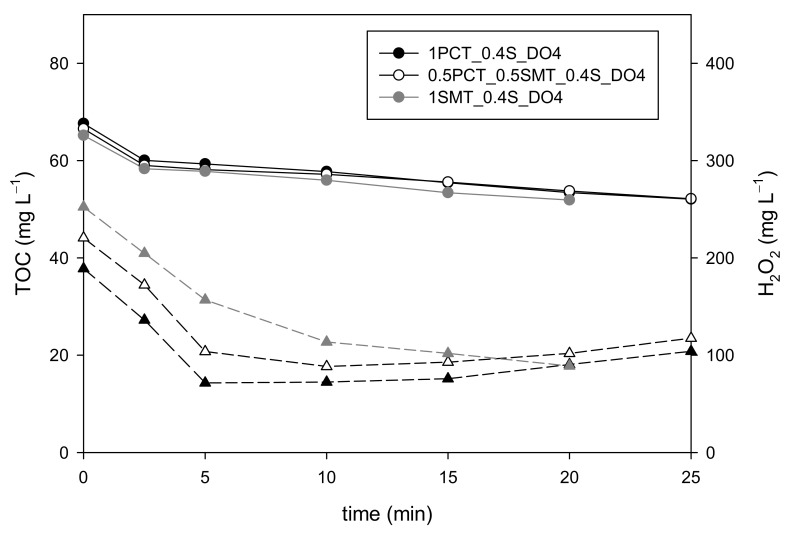
TOC (round symbols) and H_2_O_2_ (triangular symbols) concentration curves obtained with the 0.4S_DO4 dosage strategy when treating a 0.5PCT_0.5SMT mixture (63 mg of TOC L^−1^).

The results for this new assay showed that H_2_O_2_ concentration decreased from 220 mg L^−1^ to 88 mg L^−1^ in 10 min. Then, it increased up to ≈100 mg L^−1^ until DO reached 4 mg L^−1^ after 19 min (Figure 4). Therefore, the 0.4S_DO4 strategy was once again validated. Due to the interesting results regarding the use of DO slope as set point for the transition stage obtained in previous sections, the evolution of H_2_O_2_ concentration with respect to this variable was then monitored. When the DO slope reached 0.2 mg L^−1^ min^−1^ (11 min), the H_2_O_2_ concentration was 90 mg L^−1^, highlighting once again that this variable could be an interesting alternative to DO also for the transition stage.

#### 3.3.2. Sulfamethazine Contaminated Wastewater

The next set of assays was performed using only SMT (63 mg of TOC L^−---−1^), for which the three stages of the H_2_O_2_ dosage strategy were completely re-evaluated.

**Figure 5:**
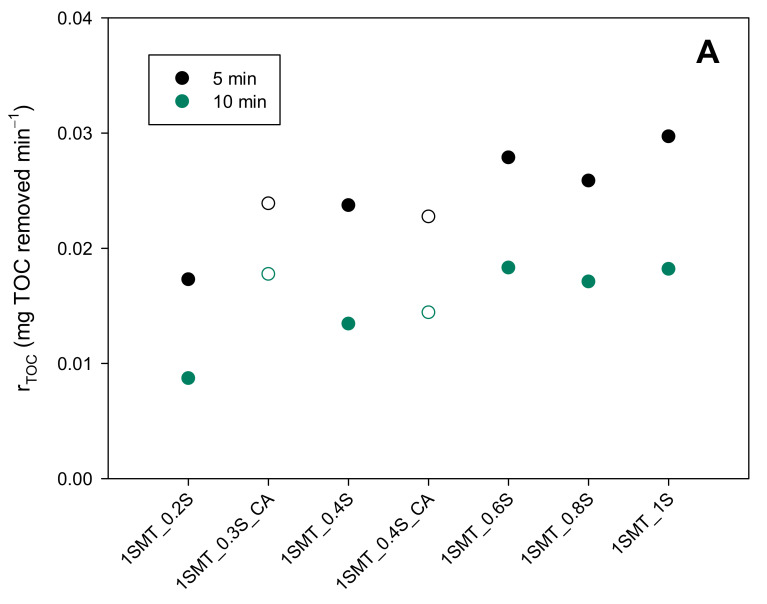

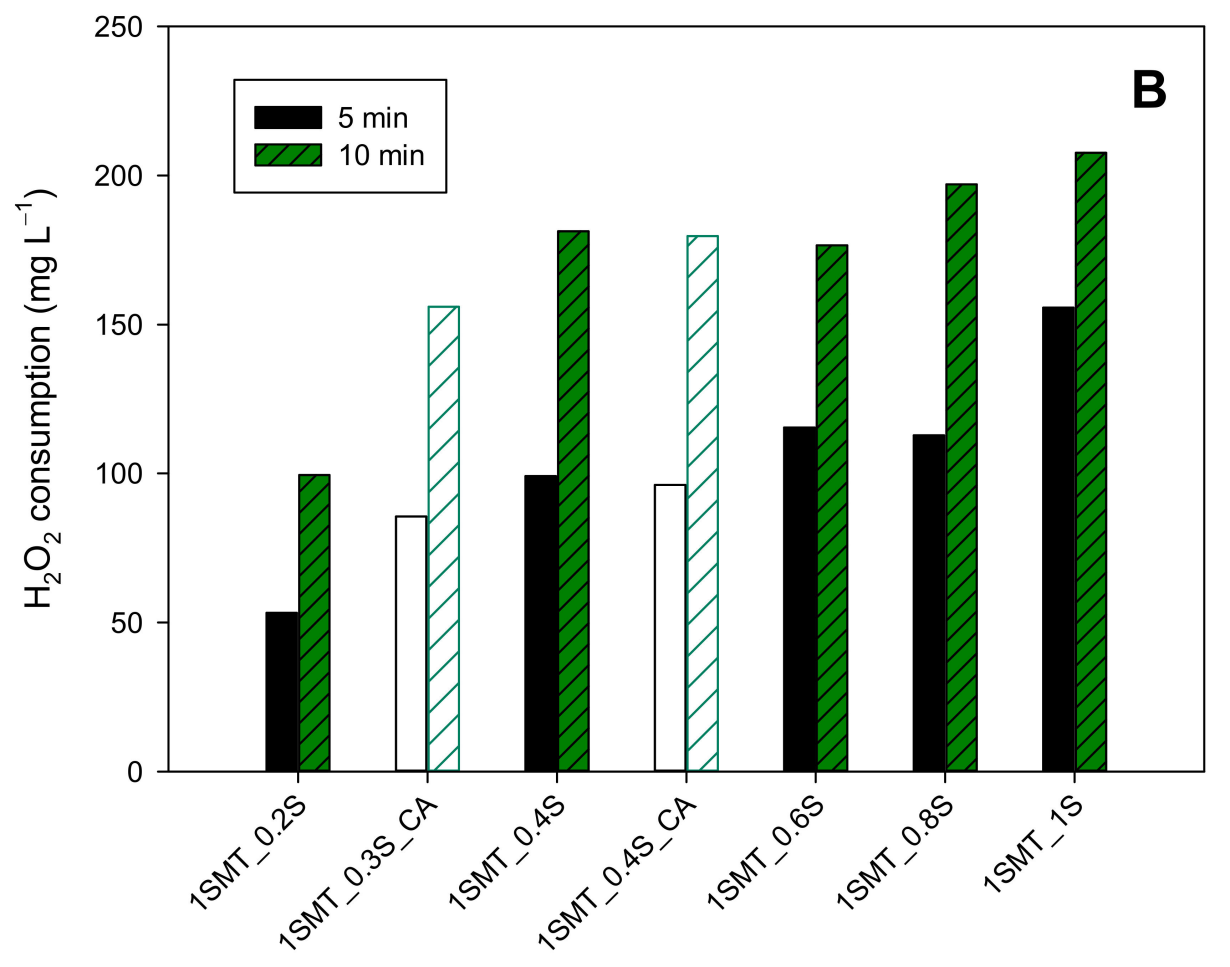
Mineralization rates (**A**) and H_2_O_2_ consumption (**B**) after 5 and 10 min of reaction time obtained for the treatment of SMT contaminated water under different initial H_2_O_2_ addition profiles (closed symbols: one-shot additions, open symbols: one-shot addition + continuous addition).

The process performance under different one-shot initial additions from 0.2S to 1S (126 mg L^−1^–631 mg L^−1^) was first evaluated (Figure 5A). Only the 1SMT_0.2S experiment presented a lower TOC reduction rate (mg L^−1^ min^−1^) than the rest of assays, which showed equivalent values. With respect to the H_2_O_2_ consumption (Figure 5B), it increased with the amount of the initial addition. While 176 mg L^−1^ were consumed in the 1SMT_0.4S assay after 10 min, this value increased up to 208 mg L^−1^ for the 1SMT_1S assay. In addition, the H_2_O_2_ concentration in the reaction bulk after 10 min for the 1SMT_0.4S assay was 71 mg L^−1^, in the middle of the desired range to minimize side reactions, whilst the H_2_O_2_ concentration after 10 min for the rest of the experiments was above 250 mg L^−1^. Thus, the 1SMT_0.4S (252 mg L^−1^) option resulted in the best alternative.

The transition stage involves the continuous addition of H_2_O_2_ from the beginning of the treatment, and this somehow also influences the initial stage, therefore, the 1SMT_0.4S approach was then compared to 0.4S and 0.3S one-shot initial additions followed by continuous addition from the beginning of the assay (codified as 1SMT_0.4S_CA and 1SMT_0.3S_CA, respectively). There were no significant differences between the different strategies up to 10 min of reaction time, hence, all three options were evaluated for the transition stage, including the 1SMT_0.4S one-shot initial addition without further continuous addition.

**Figure 6:**
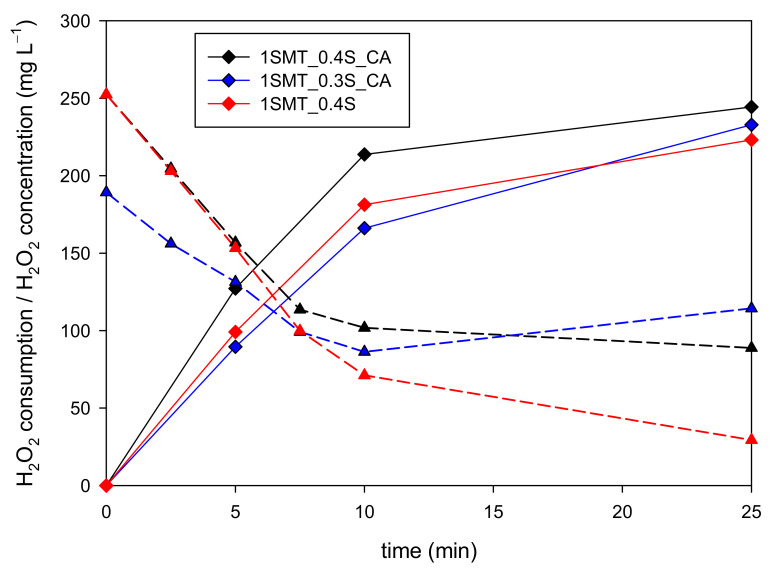
Comparison of H_2_O_2_ consumptions (continuous lines) and concentration (dashed lines) profiles obtained with the 1SMT_0.4S_CA, SMT_0.3S_CA and 1SMT_0.4S dosage strategies during the transition stage of SMT contaminated water treatment.

During the transition stage, the mineralization rates were the same independently of the selected strategy up to the moment in which each assay reached the DO set point (4 mg L^−1^). In this way, the H_2_O_2_ evolution and consumption were the variables compared to determine the feasibility of the different alternatives. Figure 6 shows that the H_2_O_2_ consumption was equivalent up to 20 min for the 1SMT_0.4S and 1SMT_0.3S_CA experiments. Moreover, the H_2_O_2_ concentration in the reaction bulk after 10 min was quite similar and within the objective range in both cases, 71 and 86 mg L^−1^, respectively. Regarding the 1SMT_0.4S_CA strategy, there was a moderate increase of H_2_O_2_ consumption with respect to the other two options after 10 min. This is in accordance with its H_2_O_2_ evolution profile because the concentration of this reagent in the system remained above 135 mg L^−1^ from 10 to 20 min favoring the proliferation of side reactions. Consequently, the 1SMT_0.4S and the 1SMT_0.3S_CA dosage strategies presented a more adequate performance than the 1SMT_0.4S_CA strategy.

**Figure 7:**
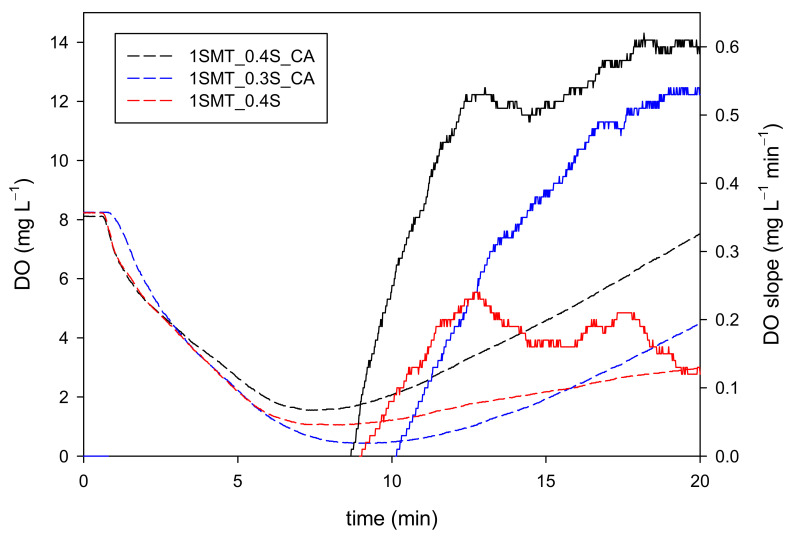
DO (dashed lines) and DO slope (lines) evolution profiles during the transition stage of SMT contaminated water treatment using 1SMT_0.4S_CA, 1SMT_0.3S_CA and 1SMT_0.4S dosage strategies.

The information provided by the DO and DO slope signals was suitable in both cases, but again more precise for the latter (Figure 7). By way of illustration, in the 1SMT_0.3S_CA experiment, the 4 mg L^−1^ DO set point was reached in 19 min, while the DO slope reached 0.2 mg L^−1^ min^−1^ in 12 min. Since the H_2_O_2_ concentration started to accumulate in the system from 86 mg L^−1^ at min 10 to >100 mg L^−1^ at min 20, to stop the continuous addition at 12 min is a more adequate response, even though 19 min is a perfectly acceptable stop time. Very similar conclusions were obtained for the rest of H_2_O_2_ dosage strategies; consequently, all the data obtained along this work suggest that DO slope provides more accurate information about H_2_O_2_ evolution than DO during the transition stage. Furthermore, the same values that have been previously used as set points for the on-off control system during the final automatic dosage stage provide the right information to stop/start the continuous addition and to mark the automatic dosage kick off. Notice that 0.1 mg L^−1^ of DO slope would mark the start of the H_2_O_2_ addition in the 1SMT_0.4S_DO4 experiment because there is no continuous addition. Thus, to integrate the transition stage and the automatic stage seems the logical option because both phases can be based on the same control variable and set points. This means a new step forward in the search for a simple and generalized H_2_O_2_ dosage strategy.

**Figure 8:**
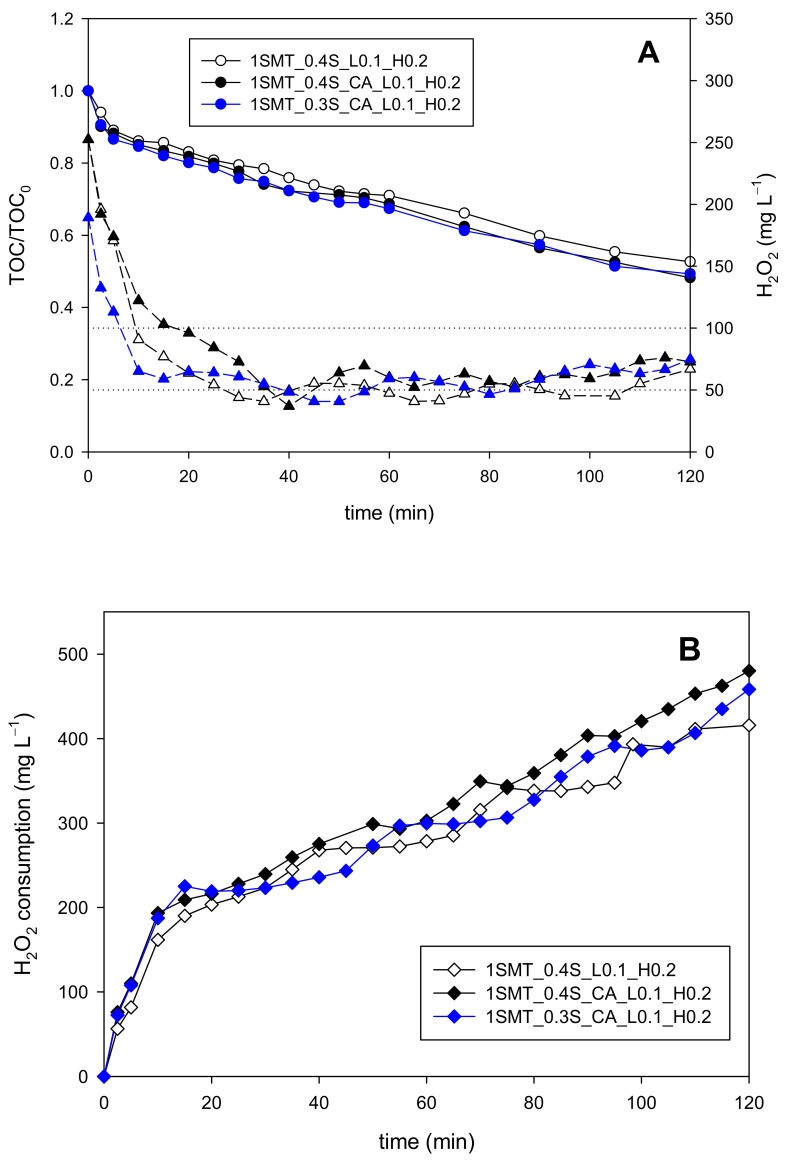
Mineralization (round symbols) and H_2_O_2_ (triangular symbols) evolution (**A**) and H_2_O_2_ consumption profiles (**B**) obtained with the 1SMT_0.4S_L0.1_H0.2, 1SMT_0.3S_CA_L0.1_H0.2 and 1SMT_0.4S_CA_L0.1_H0.2 strategies.

In this context, the last set of experiments (codification presented in Table 2) was focused on the direct coupling of the selected initial dosage strategies with the on-off control system using 0.1 mg L^−1^ min^−1^ and 0.2 mg L^−1^ min^−1^ DO slope values as set points, as explained in previous sections (1SMT_0.4S_L0.1_H0.2, 1SMT_0.3S_CA_L0.1_H0.2 and 1SMT_0.4S_CA_L0.1_H0.2). Although some small differences between the assays can be observed (Figure 8), the results of all three options were suitable with nearly equivalent efficiencies. The 0.4S_L0.1_H0.2 strategy showed a slightly lower mineralization rate (0.25 mg of TOC removed min^−1^) than the other two experiments, but also a lower H_2_O_2_ consumption (416 mg L^−1^). The opposite situation was found in the 1SMT_0.3S_CA_L0.1_H0.2 and 1SMT_0.4S_CA_L0.1_H0.2 assays, as the mineralization rates were slightly higher (0.27 and 0.26 mg of TOC removed min^−1^, respectively) but the H_2_O_2_ consumptions were also higher (458 and 480 mg L^−1^, respectively). The average H_2_O_2_ concentrations in the system from 15 min to 120 min were 52 ± 8 mg L^−1^, 58 ± 9 mg L^−1^ and 66 ± 15 mg L^−1^ for the 1SMT_0.4S_L0.1_H0.2, 1SMT_0.3S_CA_L0.1_H0.2 and 1SMT_0.4S_CA_L0.1_H0.2 assays, respectively. This indicates the ability of the control system for maintaining the H_2_O_2_ concentration within the desired range using only the DO slope as control variable and under different dosage strategies.

## 4. Discussion

The discussion section discusses the interpretation of these results in regard of possible justification mechanisms and the implications in regard to the selection of an efficient control strategy for the dosage of hydrogen peroxide.

### 4.1. Water Matrix Effect on Dosage Strategy: Distilled Water vs. Natural Water

The results revealed that the most adequate initial addition is the same for both water matrixes, NW and DW, concretely 40% of the theoretical stoichiometric concentration (denoted as 0.4S), since this was the minimum concentration that allowed obtaining the highest mineralization rate. Increasing the H_2_O_2_ initial addition above 0.4S did not improve the mineralization rate.

On the other hand, the initial oxygen consumption can be explained by the Dorfman reaction, which generates less reactive oxygen species (ROS) [16,23]. Probably, the Dorfman mechanism is boosted by the higher amount of ROS participating in the reaction mechanisms due to the higher inorganic load; thus, oxygen can be recovered earlier.

During the on-off control stage, the average H_2_O_2_ concentrations in the reaction bulk obtained were (79.8 ± 17 mg L^−1^ and 76.8 ± 12 mg L^−1^ for DW and NW, respectively, being both values practically equivalent and within the range (50–100 mg L^−1^) that allows minimizing the side reactions unrelated to organic matter mineralization [25].

The final H_2_O_2_ consumptions were 464 mg L^−1^ and 482 mg L^−1^ for DW and NW, respectively. These results indicate again that the inorganic components of the water matrix may be responsible for the slight increase of the oxidant agent consumption. The comparison between the DW and NW complete experiments presented in Figure 2 reveals that the proposed dosage strategy perfectly suits the new water matrix characteristics, which means a step forward in looking for a generalized H_2_O_2_ dosage solution. Obviously, further actions are still needed, and future studies should consider the treatment of simulated and/or actual industrial wastewaters. In any case, based on these results, a fine tuning of the dosage strategy will probably be enough to further reduce H_2_O_2_ consumption.

### 4.2. Validating the Dosage Strategy with Different Pollutant Concentrations

The drawbacks of the H_2_O_2_ dosage strategy arise from an excess of H_2_O_2_ on the initial load and/or the subsequent continuous addition, which causes H_2_O_2_ concentration to fall outside the desired concentration range favoring oxygen production instead of organic matter degradation. This is exactly what happened in the experiments in which the initial PCT concentration was increased. Since the reference dosage strategy (0.4S_DO4_L0.1_H0.2) considers the H_2_O_2_ initial addition as a function of the stoichiometric concentration, increasing the pollutant concentration resulted in H_2_O_2_ excess in the reaction bulk. In this context, the main factor determining the initial H_2_O_2_ consumption is the Fenton reaction, which involves the reaction between iron and H_2_O_2_. This is the reason why two new alternatives were evaluated: (i) to reduce the initial one-shot addition of H_2_O_2,_ and (ii) to increase the iron concentration.

Regarding the first option, the results of the 2PCT_0.3S_DO4 and 2PCT_0.25S_DO4 dosage strategies (find complete description in Table 2) allow for the comparing and evaluating of the modification of the initial one-shot addition (Figure 3), revealing that mineralization and H_2_O_2_ consumption (Figure 3B) are slightly increased with the amount of the initial addition. As previously stated, the H_2_O_2_ concentration is always above 100 mg L^−1^ when selecting the 0.4S_DO4 strategy and, although the mineralization rate is high, the more the H_2_O_2_ concentration deviates above the proposed concentration range (50–100 mg L^−1^) the more the inefficient consumption of H_2_O_2_ is favored due to the propagation of side reactions that result in dissolved oxygen concentration increase. The opposite situation is found for the 2PCT_0.25S_DO4 alternative. In this case, the H_2_O_2_ concentration after 5 min of reaction time decreased to 30 mg L^−1^, which falls below the lower limit of the desired range. This agrees with a reduction of the mineralization rate. The fact that the 2PCT_0.3S_DO4 strategy was the most adequate option may be justified because it balances the mineralization rate with respect to the total H_2_O_2_ addition. In any case, all three experiments presented similar efficiencies (mg of TOC removed per mg of H_2_O_2_ consumed) despite the differences found in the H_2_O_2_ concentration evolution during the experiments (Table 3).

Consequently, small deviations during a short time (a few minutes) can be assumed not to significantly affect the process efficiency. These results have shown that 40% of the H_2_O_2_ stoichiometric amount seems a good initial guess, although the value is subject to tuning, especially for highly contaminated wastewaters. Adjusting this value should consider that the H_2_O_2_ concentration during the first minutes (mainly driven by Fenton reaction) should already be within the 50–100 mg L^−1^ range.

Interestingly, the comparison of the results of these three assays (Table 3) reveals that the DO slope, proposed to be used only for the final stage of the dosage strategy, could also be selected as the control variable at the transition stage. For instance, using the DO slope high threshold of the on-off control system (0.2 mg L^−1^ min^−1^) would allow stopping the dosage of the transition stage after 31 min in the 2PCT_0.4S_DO4 assay, about 8 min earlier than when using 4 mg L^−1^ of DO as set up. The same efficiency would be obtained (0.06 mg TOC mg H_2_O_2_^−1^) with less time (20% approx.) and lower H_2_O_2_ consumption (8% approx.). Therefore, it is worth noting that adopting the DO slope signal (0.2 mg L^−1^ min^−1^) instead of DO as the control variable in the transition stage allows for the improving of the process performance, even though selecting 4 mg L^−1^ of DO still provides an acceptable response.

Regarding the other alternative, the iron concentration was increased proportionally to the initial pollutant concentration, i.e., iron was doubled from 20 mg L^−1^ to 40 mg L^−1^ (2PCT_0.4S_DO4_2Fe). Obviously, increasing iron concentration increases H_2_O_2_ consumption, which results in the higher production of radicals. However, the results obtained need further rationalization. Increasing iron concentration should be carefully balanced against the possibility of radiation becoming limiting. In this case, the theoretical limit for the iron concentration in the photo-reactor is 40 mg L^−1^ [26] and higher concentrations would have no further effect on the process outcome during the illuminated phase of the treatment. The key point is to find out if this theoretical higher availability of radicals due to the higher iron concentration is translated into organic matter oxidation reactions.

Figure 3 again reveals that mineralization was improved with respect to the previously described experiments. Thus, the H_2_O_2_ concentration in the reaction bulk could be kept within the 50–100 mg L^−1^ range from 5 min of reaction time up to the moment in which the DO set point was reached. This option was also validated with an intermediate concentration of PCT (1.5PCT, i.e., 150 mg L^−1^ of the compound equivalent to 95 mg L^−1^ of TOC), with identical results to the ones described in this section (data not shown). The results also showed that both the 4 mg L^−1^ DO and the 0.2 mg L^−1^ min^−1^ DO slope set points could be selected as control signals for the transition stage. In terms of efficiency, increasing the iron concentration presented values equivalent to those obtained for the initial dosage modification alternative (Table 3).

**Figure 9:**
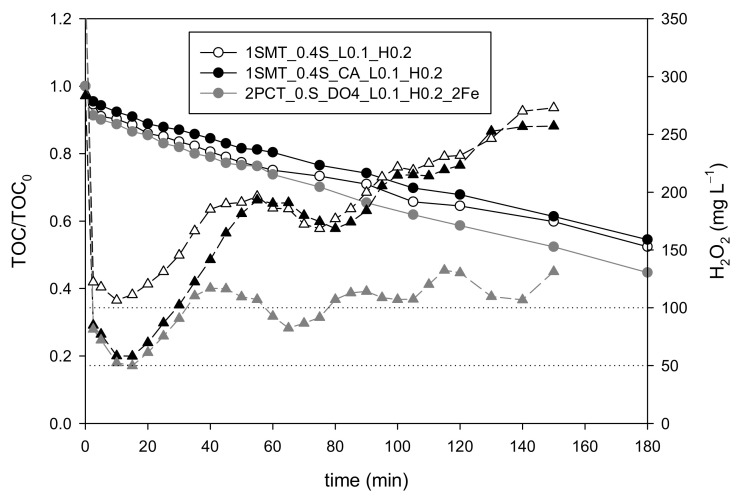
Comparison of different complete H_2_O_2_ dosage strategies for the treatment of 2PCT contaminated wastewater (126 mg L^−1^ TOC) by photo-Fenton process (TOC and H_2_O_2_ concentration profiles are represented by round and triangular symbols, respectively).

Finally, three assays considering the complete dosage strategy (all three steps) can be similarly compared: 2PCT_0.4S_DO4_L0.1_H0.2, 2PCT_0.3S_DO4_L0.1_H0.2 and 2PCT_0.4S_DO4_L0.1_H0.2_2Fe (codification presented in Table 2). The results (Figure 9) reveal that preserving the iron to contaminant ratio allows keeping H_2_O_2_ concentration much closer to the desired range 50-100 mg L^−1^ than the rest of alternatives. As expected, the H_2_O_2_ concentration in the 2PCT_0.4S_DO4_L0.1_H0.2 experiment deviated from the desired range from the beginning of the assay. The main deviation in the 2PCT_0.3S_DO4_L0.1_H0.2 experiment was an accumulation of H_2_O_2_ when the on-off control system was applied, which means that the DO slope thresholds need to be tuned for their adaption to the new wastewater characteristics. Notice that the initial H_2_O_2_ addition was already modified with respect to the base strategy.

**Figure 10:**
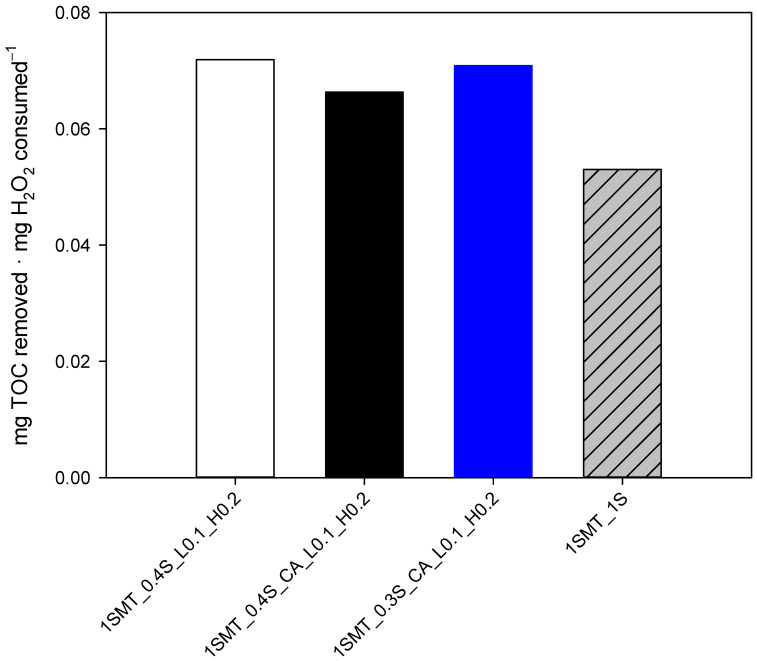
Comparison of the photo-Fenton process efficiency in terms of mg of TOC removed per mg of H_2_O_2_ consumed for each dosage strategy, including the one-shot initial addition of the theoretical stoichiometric concentration.

If the results of sulfamethazine contaminated wastewater are translated into efficiencies, then the 1SMT_0.4S_L0.1_H0.2 and 1SMT_0.3S_CA_L0.1_H0.2 dosage strategies (Table 2) present the highest values, 0.72 and 0.71 mg of TOC removed per mg of H_2_O_2_ consumed, respectively, followed closely by the 1SMT_0.4S_CA_L0.1_H0.2 assay that exhibited 0.67 mg of TOC removed per mg of H_2_O_2_ consumed. In any case, as shown in Figure 10, these values represent 25–35% improvement with respect to the efficiency obtained in the 1S assay (one-shot initial addition of the theoretical stoichiometric concentration) validating the simplified dosage strategies. This involves a significant reduction of treatment cost. By way of illustration, considering the daily treatment of 100 m^3^ of polluted water and 0.5€/kg as the H_2_O_2_ unitary price [27], ≈3500€ annually in operating costs could be saved by implementing the 0.4S_CA_L0.1_H0.2 dosage strategy with respect to the single addition strategy. Furthermore, the implementation of controlled additions of H_2_O_2_ represents a substantial step forward for process automation, which is obviously a critical factor for the photo-Fenton process achieving full-scale applications.

## 5. Conclusions

This work represents a step forward in the development of a generalized scheme for H_2_O_2_ dosage in photo-Fenton processes. The work has shown that DO monitoring (a process variable that is inexpensively measured) enables the design of efficient dosage strategies. These strategies were validated against different compounds and mixtures, providing a new insight into this dosage methodology. Hence, the dosage strategy has been redesigned into a simpler and more robust scheme. Despite the different nature of the pollutants studied, the results obtained for PCT, SMT and their mixtures have proved the dosage strategy to be general enough to produce similar degradation rates for the same organic load (TOC). Outcomes have also demonstrated that the dosage strategy could address different organic loads (double concentration of PCT and SMT mixtures) by applying minor modifications (fine tuning). These findings allowed for the improving of the treatment efficiency in terms of TOC removed per H_2_O_2_ consumed in the range from 8 to 15%, which would lead to a significant reduction of operating costs. The implementation of this dosage strategy is also a step forward in process automation, with a substantial positive impact on photo-Fenton TRL (technology readiness level).

The DO slope, which was previously used only for driving the on-off control system of the last treatment stage, was also found to be useful at the transition stage by reducing its duration while achieving the same outcome. This is important, since after the initial addition of H_2_O_2_ shared by both options, the original conception combining open-loop and closed-loop dosage control was tested against the use of a closed-loop dosage control system only, which obtained similar results for both contaminants, PCT, SMT and the mixtures.

Further research is underway to explore the tuning of this new dosage scheme beyond the current limiting assumptions. The on-off control has been established according to the range of H_2_O_2_ concentration values recommended in the literature (50–100 mg L^−1^). Nevertheless, this recommendation does not contemplate the feedback that the DO slope can provide, and this range could be also tuned for further increases to the efficiency of the dosage, for instance by reducing it as the treatment goal approaches.

## Figures and Tables

**Table 1:** Photoreactor specifications.

Irradiated volume, L		1.5
Annular irradiated height, mm		130
Outer cylinder	Outer diameter, mm	150
	Inner diameter, mm	140
Inner cylinder	Outer diameter, mm	70
	Inner diameter, mm	63.6

**Table 2:** Experimental plan.

Assay Code	PCT	SMT	TOC	Iron	Initial H_2_O_2_	Dosage Stage		
	mg L^−1^	mg L^−1^	mg L^−1^	mg L^−1^	mg L^−1^	Initial	Transition	Final
1PCT_0.4S_DO4_L0.15_H0.25_DW	100	0	63	20	189	YES	YES	YES
1PCT_0.4S_DO4_L0.15_H0.25_NW	100	0	63	20	189	YES	YES	YES
2PCT_0.25S_DO4	200	0	126	20	236	YES	YES	NO
2PCT_0.3S_DO4	200	0	126	20	283	YES	YES	NO
2PCT_0.4S_DO4	200	0	126	20	378	YES	YES	NO
2PCT_0.4S_DO4_2Fe	200	0	126	40	378	YES	YES	NO
2PCT_0.3S_DO4_L0.1_H0.2	200	0	126	20	283	YES	YES	YES
2PCT_0.4S_DO4_L0.1_H0.2	200	0	126	20	378	YES	YES	YES
2PCT_0.4S_DO4_L0.1_H0.2_2Fe	200	0	126	40	378	YES	YES	YES
1PCT_0.4S_DO4	100	0	63	20	189	YES	YES	NO
1SMT_0.4S_DO4	0	123	63	20	252	YES	YES	NO
0.5PCT_0.5SMT_0.4S_DO4	50	61.5	63	20	221	YES	YES	NO
1SMT_0.2S	0	123	63	20	126	YES	NO	NO
1SMT_0.3S_CA	0	123	63	20	189	YES	YES	NO
1SMT_0.4S	0	123	63	20	252	YES	NO	NO
1SMT_0.4S_CA	0	123	63	20	252	YES	YES	NO
1SMT_0.6S	0	123	63	20	379	YES	NO	NO
1SMT_0.8S	0	123	63	20	505	YES	NO	NO
1SMT_1.0S	0	123	63	20	631	YES	NO	NO
1SMT_0.4S_CA_L0.1_H0.2	0	123	63	20	252	YES	NO	YES
1SMT_0.3S_CA_L0.1_H0.2	0	123	63	20	189	YES	NO	YES
1SMT_0.4S_L0.1_H0.2	0	123	63	20	252	YES	NO	YES

**Table 3:** Information for each dosage strategy studied about H_2_O_2_ addition/evolution, reaction time and efficiency needed to reach the proposed set points when PCT concentration was doubled up to 126 mg L^−1^ TOC.

		Dosage Strategy			
		2PCT_0.25S_DO4	2PCT_0.3S_DO4	2PCT_0.4S_DO4	2PCT_0.4S_DO4_2Fe
Initial H_2_O_2_ concentration (mg L^−1^)		236	283	377	377
Minimum H_2_O_2_ concentration in reaction bulk (mg L^−1^)		30	63	118	46
DO set point (4 mg L^−1^)	time (min)	55	47	39	36
	Equivalent* H_2_O_2_ concentration (mg L^−1^)	583	580	623	604
	Actual H_2_O_2_ concentration (mg L^−1^)	169	159	184	106
	Efficiency (mg TOC mg H_2_O_2_^−1^)	0.06	0.06	0.06	0.06
DO slope set point (0.2 mg L^−1^ min^−1^)	time (min)	47	34	31	26
	Equivalent * H_2_O_2_ concentration (mg L^−1^)	533	498	573	541
	Actual H_2_O_2_ concentration (mg L^−1^)	125	114	157	75
	Efficiency (mg TOC mg H_2_O_2_^−1^)	0.06	0.05	0.06	0.06

* The equivalent concentration is defined as the one that would result from the addition of the same amount of H_2_O_2_ to a non-reacting system (no consumption of H_2_O_2_).
